# Determination of Optimum Viewing Angles for the Angular Normalization of Land Surface Temperature over Vegetated Surface

**DOI:** 10.3390/s150407537

**Published:** 2015-03-27

**Authors:** Huazhong Ren, Guangjian Yan, Rongyuan Liu, Zhao-Liang Li, Qiming Qin, Françoise Nerry, Qiang Liu

**Affiliations:** 1 State Key Laboratory of Remote Sensing Science, School of Geography, Beijing Normal University, Beijing 100875, China; E-Mail: renhuazhong@mail.bnu.edu.cn; 2 Institute of Remote Sensing and Geographic Information System, Peking University, Beijing 100871, China; E-Mail: qmqinpku@163.com; 3 Key Laboratory of Agri-Informatics, Ministry of Agriculture/Institute of Agricultural Resources and Regional Planning, Chinese Academy of Agricultural Sciences, Beijing 100081, China; E-Mail: lizhaoliang@caas.cn; 4 ICube Laboratory, Université de Strasbourg, 67412 Illkirch, France; E-Mail: f.nerry@unistra.fr; 5 College of Global Change and Earth System Science, Beijing Normal University, Beijing 100875, China; E-Mail: toliuqiang@bnu.edu.cn

**Keywords:** BRDF, angular normalization, land surface temperature, multi-angular, WiDAS

## Abstract

Multi-angular observation of land surface thermal radiation is considered to be a promising method of performing the angular normalization of land surface temperature (LST) retrieved from remote sensing data. This paper focuses on an investigation of the minimum requirements of viewing angles to perform such normalizations on LST. The normally kernel-driven bi-directional reflectance distribution function (BRDF) is first extended to the thermal infrared (TIR) domain as TIR-BRDF model, and its uncertainty is shown to be less than 0.3 K when used to fit the hemispheric directional thermal radiation. A local optimum three-angle combination is found and verified using the TIR-BRDF model based on two patterns: the single-point pattern and the linear-array pattern. The TIR-BRDF is applied to an airborne multi-angular dataset to retrieve LST at nadir (*T_e_*-*nadir*) from different viewing directions, and the results show that this model can obtain reliable *T_e_*-*nadir* from 3 to 4 directional observations with large angle intervals, thus corresponding to large temperature angular variations. The *T_e_*-*nadir* is generally larger than temperature of the slant direction, with a difference of approximately 0.5~2.0 K for vegetated pixels and up to several Kelvins for non-vegetated pixels. The findings of this paper will facilitate the future development of multi-angular thermal infrared sensors.

## 1. Introduction

Land surface temperature (LST) is required in many applications, including agrometeorology, climate and environmental studies [1,2]. Thermal infrared images from aircraft and spaceborne satellites provide a unique opportunity to map this parameter at regional and even global scales. Many methods have been proposed to retrieve LST data from remotely sensed data using different specifications of thermal infrared sensors and the atmospheric and emissivity data situations [3,4,5]. These methods can been roughly grouped into three categories: single-channel algorithms [6,7], multi-channel methods (e.g., the split-window algorithm [8,9] and the temperature and emissivity separation method [10,11]), and multi-time methods (e.g., the temperature-independent spectral index (TISI) method [12,13], the two-temperature method (TTM) [14], and the physical day/night algorithm [15]). For operational purposes, these methods often take the observed pixel as a homogeneous and isothermal target; this assumption is reasonable for pure or quasi-pure pixels such as bare soil, sand, snow and dense vegetated surfaces. However, for mixed pixels including two or more components at different temperatures and emissivities, their pixel temperature exhibits spectral and angular variations. As a result, the above assumption will be incorrect, and the retrieved LST will only present, at the least, the effective temperature at its corresponding viewing direction and cannot be directly taken as the temperature at nadir or under other directions in theory.

The angular behavior of LST has been investigated in many previous studies [16,17,18,19,20,21,22], and this angular variation results primarily from the angular variation in the pixel emissivity for three-dimensional surfaces and the relative weights of more than one component (e.g., leaf and background soil) with different temperatures included in the scene. Certain ground measurements have indicated that the LST difference at nadir and off-nadir observations can be as large as 5 K for bare soils and up to 10 K for urban areas [23]. For satellite images, the pixels also face a similar situation because the pixels in the same image are sometimes observed at different viewing angles, especially by those sensors with a large field of view (FOV). For example, the Moderate Resolution Imaging Spectroradiometer (MODIS) scans the land surface in the cross-track direction with a viewing zenith angle (VZA) varying from −65° to +65° [21,24]. Thus, angle-dependent variations in the retrieved LST are inevitable, which make the LSTs of different pixels in the same image incomparable and eventually lead to an error up to about 2.5 K [21]. Similar cases can be observed in other satellite sensors such as the Advanced Very High Resolution Radiometer (AVHRR) and Spinning Enhanced Visible and InfraRed Imager (SEVIRI) instruments. Therefore, it is crucial to perform angular normalization of the LST data, *i.e.*, to translate LST data obtained in different directions to a specified direction such as the nadir direction [3,25].

Multi-angular observation of the same target is considered to be the most promising method of solving this problem, and such observations can be achieved using ground goniometers: the Portable Apparatus for Rapid Acquisitions of Bi-directional Observations of Land and Atmosphere (PARABOLA) [26], the field goniometer system (FIGOS) [27], the portable Multi-Angle Observation System (MAOS) [23,28], and others [29,30]. However, only a small number of studies have been published on LST angular normalization or on the simultaneous retrieval of directional emissivity and temperature from multi-angular TIR images mainly due to a lack of multi-angular observations at satellite level. To date, only ATSR (Advanced Along Track Scanning Radiometer) series satellites [31,32] have provided multi-angular observations of thermal infrared (TIR) data because of the difficulty and the complexity of manufacturing highly controlled multi-angular sensors and also because no studies that discuss requirements in terms of the number of direction specifications of angular observations have been published. To address this situation, we investigate the minimum requirements of viewing observations and directions for achieving angular normalization of LSTs using multi-angular TIR data to provide technique support for the future design of such sensors. This paper is organized as follows: Section 2 will introduce a new model to simulate the directional thermal radiance and discuss the performance of the kernel-driven bi-directional reflectance distribution function (BRDF) model in the TIR domain as a TIR-BRDF model. Section 3 will investigate the local optimum three-angle combinations for the TIR-BRDF using a single-point pattern and discuss the three-angle combinations using a linear array pattern that is always used in airborne and satellite sensors. Section 4 will apply the TIR-BRDF model to an airborne multi-angular TIR image from the WiDAS (Wide-angle infrared Dual-mode line/area Array Scanner) system [22,33] in the WATER (Watershed Allied Telemetry Experimental Research) campaign [34]. Section 5 and Section 6 will provide discussions and conclusions of this paper. The full names and the corresponding abbreviations of some terms are listed in the Table A1 of Appendix.

## 2. Modeling of Directional Thermal Radiation

Modeling the directional thermal radiance (DTR) of homogenous or heterogeneous canopies has attracted a substantial amount of attention. The main reason for the DTR is that the fractions of different components with different temperature and emissivity vary with the changes of viewing angles. Based on this concept, considering *N* different components in a pixel under clear-sky conditions, a generalized parameterization of the DTR can be expressed as [22]:

(1) L(θv,θs,ϕ)=B[DBT(θv,θs,ϕ)]=ε(θv,ϕv)B[Te(θv,θs,ϕ)]                  =∑i=1Nfi(θv,θs,ϕ)⋅εi⋅Bi(Ti)+Lmulti

where θ*_v_* and θ*_s_* are the viewing zenith angle (VZA) and the solar zenith angle (SZA), respectively; φ is the relative azimuth angle (RAA) between the viewing azimuth angle (VAA) φ*_v_* and the solar azimuth angle (SAA) φ*_s_*; *L*(θ*_v_*, θ*_s_*, φ) is the directional thermal radiance (the inversion of this term according to the Planck’s law *B*[] will produce the directional brightness temperature (DBT)); Meanwhile, ε(θ*_v_*,φ*_v_*) is the pixel ensemble directional emissivity and *T_e_*(θ*_v_*, θ*_s_*, φ) is the directional effective temperature; *f_i_* are the fractions of various components, such as the commonly used sunlit and shadowed leaves and sunlit and shadowed soil; *T_i_* and ε*_i_* are the temperatures (unit: K) and emissivities of those components, and note that ε*_i_* are assumed to be independent of viewing angle; and *L_multi_* is the multi-scattering between soil and leaves and between leaves inside the canopy.

We consider the homogenous canopy and estimate the component fractions *f_i_* from a parameterization [35] of the Scattering by Arbitrarily Inclined Leaves (SAIL) Hotspot (SAILH) model [36,37] using the solar and viewing directions, canopy structure and model parameters. In addition, only three components (leaves and sunlit and shadowed soils) are considered because the temperature difference between sunlit and shadowed leaves is very small compared to the temperature difference between sunlit and shadowed soils. Furthermore, we calculate the multi-scattering term *L_multi_* with the following equation:

(2) $$L_{multi}=(1-M)L_{leaf}(1-\varepsilon_{g})+(1-\alpha )[1-b(\theta )M]\cdot [1-b(\theta )](1-\varepsilon_{v})\cdot L_{leaf}$$

The first part of the right-hand side of Equation (2) is the downward leaves’ radiation reflected by the soil, and the second part is the multi-scattering interior to the canopy; ε_g_ and ε_v_ are the emissivities of ground soil and leaf, respectively; *b*(θ) is the directional gap frequency in the θ direction; and *M* is the hemispheric gap probability of the canopy. For a canopy with a spherical leaf angle distribution and random dispersion, *b*(θ) and *M* can be expressed as Equation (3), in which LAI is the leaf area index. α denotes the cavity effect that accounts for multiple scatterings inside the canopy, the details of which can be found in [38].

(3) $$b(\theta )=\exp[-\frac{0.5}{\cos\theta }LAI],\;\text{and}\;M=\frac{1}{\pi }\int_{-\pi /2}^{\pi /2}b(\theta )d\theta =\exp(-0.825LAI)$$

Table 1 lists the main input variables of the simulation DBT, including the solar zenith and azimuth angles; LAI of the canopy; the temperatures of the leaves (*T_leaf_*), sunlit soil (*T_sun_soil_*) and shadowed soil (*T_shd_soil_*); and the emissivities of leaf (ε*_v_*) and soil (ε*_g_*). Note that the simulated DBT is assumed to be atmospherically corrected.

**Table 1:** The main input parameters for the directional brightness temperature (DBT) simulation.

SAA	SZA	LAI	*T_leaf_*	*T_sun_soil_*	*T_shd_soil_*	ε *_v_*	ε *_g_*
120°	30°	0.5~5.0 with a step of 0.5	305 K	320 K	315 K	0.985	0.95

In addition, we also used the kernel-driven BRDF model, which is always used to link the bi-directional reflectance to the viewing geometry in the visible/near-infrared wavelengths and to link the DTR and DBT to the viewing geometry by replacing the bi-directional reflectivity with the DTR [22,39]. As a result, the kernel-driven BRDF in the TIR field is written as Equation (4). For simplicity, the new kernel-driven BRDF model is henceforth called TIR-BRDF.

(4) $$DTR(\theta_{v},\theta_{s},\phi )=B[DBT(\theta_{v},\theta_{s},\phi )]=f_{iso}+f_{vol}\cdot k_{vol}(\theta_{v},\theta_{s},\phi )+f_{geo}\cdot k_{geo}(\theta_{v},\theta_{s},\phi )$$

where *f_iso_* is the isotropic scattering term, *f_vol_* is the coefficient of the volumetric kernel *k_vol_*, and *f_geo_* is the coefficient of the geometric kernel *k_geo_*. The Ross-Thick kernel developed in [40] and the Li-SparseR kernel derived in [41] on the basis of the geometric-optical mutual shadowing BRDF model [42] are used as the volumetric and geometrical scattering kernels, respectively. A suitable expression of *k_vol_* was derived by Roujean *et al.* [40], called the Ross-Thick kernel for its assumption of a dense leaf canopy (see Equation (5)). It is a single-scattering solution of the radiative transfer equation for plane-parallel dense leaf canopy with uniform leaf angle distribution, a Lambertian background and equal leaf transmittance and reflectivity, but it does not account for the hotspot effect.

(5) $$k_{vol}(\theta_{v},\theta_{s},\phi )=\frac{(\pi /2-\xi )\cos\xi +\sin\xi }{\cos\theta_{v}+\cos\theta_{s}}-\frac{\pi }{4}$$

where, ξ sis the phase angle, related to the sun-target-observer position as:

(6) $$\xi =\cos\;\theta_{v}\;\cos\;\theta_{s}\;+\sin\;\theta_{v}\;\sin\;\theta_{s}\;\cos\;\phi$$

The Li-Sparse kernel derived from the geometric-optical mutual shadowing BRDF model [42] was reported to work well with the observed data. The original form of this kernel is not reciprocal in the viewing and solar directions, a property that is expected from homogeneous natural surface viewed at coarser spatial resolution, but then was refined to be reciprocal by assuming the sunlit component in the viewed scene simply varies as 1/cosθ_s_. As a result, the reciprocal model was the Li-SparseR kernel [43,44]:

(7) $$\begin{array}{l} k_{geo}(\theta_{v},\theta_{s},\phi )=O(\theta_{v},\theta_{s},\phi )-\sec\theta \prime_{v}-\sec\theta \prime_{s}\\ \;+\frac{1}{2}(1+\cos\xi \prime )\sec\theta \prime_{v}\sec\theta \prime_{s} \end{array}$$

with,

$$O=\frac{1}{\pi }(t-\sin t\cos t)(\sec\theta \prime_{v}+\sec\theta \prime_{s})$$

$$\cos t=\frac{h}{b}\frac{\sqrt{D^{2}+{\left(\tan\theta \prime_{v}\tan\theta \prime_{s}\sin\phi \right)}^{2}}}{\sec\theta \prime_{v}+\sec\theta \prime_{s}}$$

$$D=\sqrt{\tan^{2}\theta \prime_{v}+\tan^{2}\theta \prime_{s}-2\tan\theta \prime_{v}\tan\theta \prime_{s}\cos\phi }$$

$$\cos\xi \prime =\cos\theta \prime_{v}\cos\theta \prime_{s}+\sin\theta \prime_{v}\sin\theta \prime_{s}\cos\phi$$

$$\theta \prime_{v}=\tan^{-1}(\frac{b}{r}\tan\theta_{v}),\theta \prime_{s}=\tan^{-1}(\frac{b}{r}\tan\theta_{s}),$$

where, *O* is the overlap area between the view and solar shadows. The term cos*t* should be constrained to the range (−1, 1), as values outsides of this range imply no overlap and should be disregarded. The ratio *h/b* and *b/r* are the dimensionless crown relative height and shape parameters, respectively, and should be preselected. In this thesis, *h/b* = 2 and *b/r* = 1, *i.e.*, the spherical crowns are separated from ground by half their diameter.

**Figure 1:**
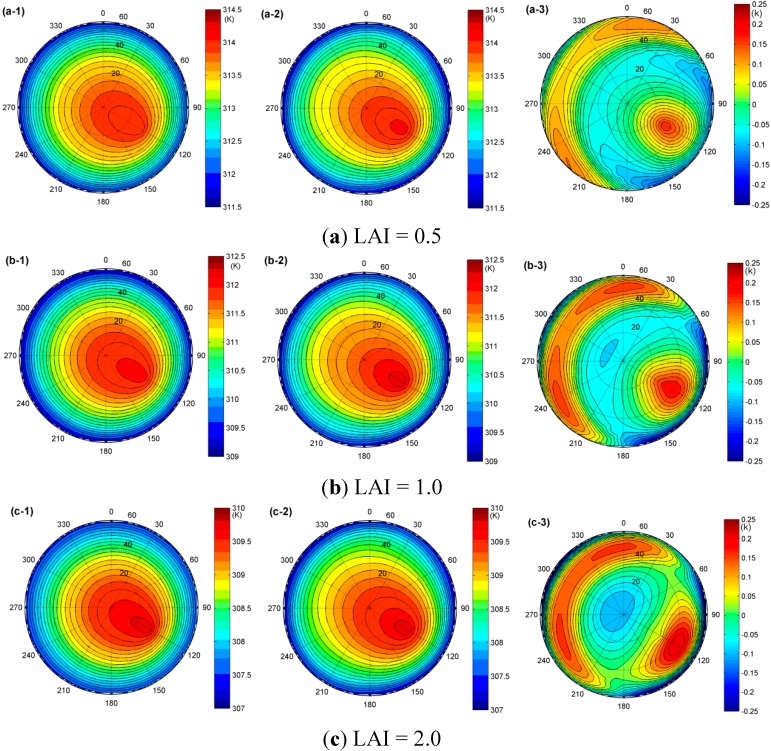
The hemispheric distribution of the simulated DBT from Equation (1) (the first column), the fitted DBT from the TIR-BRDF model (the second column) and their temperature difference (the third column) at (**a**) LAI = 0.5; (**b**) LAI = 1.0 and (**c**) LAI = 2.0, respectively.

Figure 1 shows the simulated DBT distribution (the first column), the fitted DBT from the TIR-BRDF model (the second column), and their temperature difference (the third column) at (a) LAI = 0.5; (b) LAI = 1.0 and (c) LAI = 2.0 based on the input variables shown in Table 1 for the DBT simulation using Equation (1) and the SAILH parameterization model [35]. The maximum zenith angle is constrained to 60° because the kernel-driven BRDF model was reported to obtain unacceptable results for larger zenith angles [38,45].

**Figure 2:**
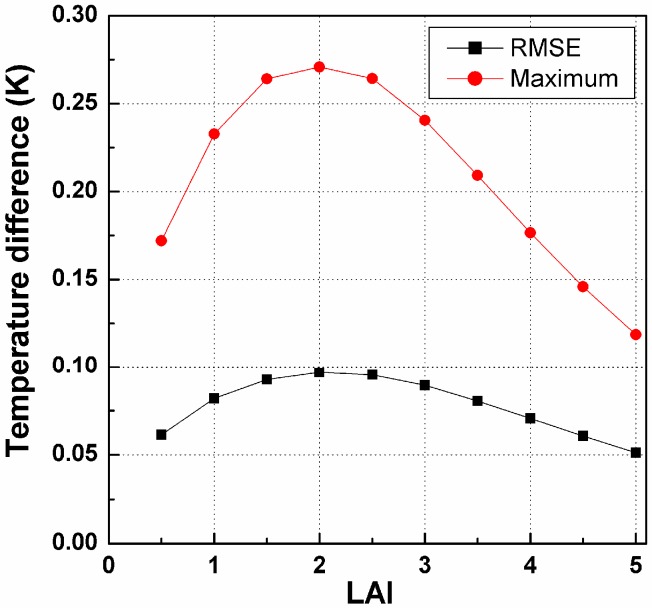
The influence of LAI on RMSE and maximum temperature difference from the TIR-BRDF model.

As observed from Figure 1, the angular variation in the DBT can be as high as approximately 3.0 K under the simulation conditions (Table 1). However, this angular variation is based on the components’ temperature difference and canopy structure: larger component temperature differences generally lead to larger angular variations. The TIR-BRDF model produces a good fit of the DBT distribution, with errors lower than 0.3 K. The hotspot effect in the solar direction is significant; however, we also find that the maximum temperature difference occurs around this direction and that the DBT of the TIR-BRDF model is generally larger than that of the simulation result in the directions around the solar beam. The increase in LAI decreases the DBT because the fraction of leaves increases and the leaves’ temperature is lower than that of the soils. Furthermore, the LAI also influences the temperature difference caused by the regression used in the TIR-BRDF model. Figure 2 shows the RMSE (root-mean-square error) and the maximum temperature difference as a function of LAI. The figure indicates that the RMSE is smaller than 0.1 K and that the maximum temperature is smaller than 0.3 K; both obtain their global maximums at approximately LAI = 2.

Furthermore, we also implemented the TIR-BRDF model using different solar positions, component temperatures and emissivities, and canopy structures, and the results further confirmed the performance of the TIR-BRDF model for calculating the DBT distribution. Note that because the component emissivities are considered to be angle independent and because, consequently, the DBT of a bare surface is isotropic, we only discuss at the level of the canopy rather than for the case of a bare surface.

## 3. Viewing Angle Specification for Angular Normalization of LST

The above result is obtained using the TIR-BRDF model with all viewing zenith and azimuth angles. However, in practice, it is almost impossible to observe the same target at numerous directions. On the contrary, only some angular observations are usually conducted. Consequently, a problem arises: how many observations and under what viewing angles are needed for the TIR-BRDF model to accurately fit the DBT and consequently enable the angular normalization of the LST? To determine the optimum observation groups, this paper used two different patterns in the following discussions: single-point patterns and linear-array patterns.

### 3.1. Single-Point Pattern Analysis for Multiple-Viewing Angle Specification

According to Equation (4), at least three angular observations are needed to drive the TIR-BRDF model. Additional observations can improve the reliability of the fitted coefficients in theory, but the errors in those observations may produce greater uncertainty in the fitted result. Therefore, we start with only three viewing angles to determine the local optimum viewing angle combination that produces the smallest error in the TIR-BRDF model and to provide various suggestions for the future development of multi-angular airborne or space-borne TIR sensors. Therefore, to better facilitate the development of a mechanical design of such a sensor, we herein assume that all observations are performed with the azimuths in the same plane, *i.e.*, their azimuths equal φ or φ + 180°.

Taking φ = 0° for example, the three observations’ zenith angles vary in the azimuth plane (0°~180°) at a step of 10° to a maximum of 60° in the zenith direction. Different observations are required to have different value of zenith or azimuth angles. As a result, there are 126 groups with three angles. To determine the optimum group, we first use Equation (1) to simulate hemispheric DBTs under varying solar positions (SAA from 0° to 330° with a step of 30°, SZA = 10°, 30° and 50°), LAIs (from 0.5 to 5.0 with a step of 0.5) and the component temperatures and emissivities in Table 1, thus resulting in a total of 396 cases. Consequently, the three coefficients in Equation (4) are obtained by regressing the DBT with the known kernels (*k_vol_* and *k_geo_*) calculated in different viewing geometry, and this process is additionally controlled by an optimization algorithm on the basis of that the brightness temperature at the hotspot direction (*i.e*., solar beam direction) has the large value because this direction corresponds to the largest proportions of sunlit soil and leaves which have a higher temperature. Table 2 shows the frequency of the temperature RMSEs in the ranges of [0.0, 0.5] K and [0.5, 1.0] K and the number of times the maximum and minimum RMSEs are obtained for different angle groups. Furthermore, because it was found that the viewing angle group with nadir viewing direction (VZA = 0° and VAA = 0°) had better results than those without such nadir observation, and also because of space limitations of this paper, we did not display those results without the nadir observation.

Furthermore, four criteria are proposed here to filter the final optimum angle combinations:

(1) Most of the RMSEs in the TIR-BRDF model should fall in the range of 0.0~0.5 K.
(2) The maximum difference should not or seldom occur for acceptable combinations of angles.
(3) Most of the minimum differences can be obtained using the combinations.
(4) Angle difference of the adjacent viewing angles should be large enough in order to cause large difference in the component fractions and then lead a large angular variation in the observed brightness temperature. But a very large VZA are not recommended in order to avoid large pixel size differences in the angle combination.

**Table 2:** The frequency of the root-mean-square error (RMSE) in different three-direction combinations. Columns 1 and 2 are the VAA and VZA for the first direction observation, and columns 3 and 4 are for the second direction observation, and columns 5 and 6 are for the third direction observation.

1st VAA	1st VZA	2nd VAA	2nd VZA	3rd VAA	3rd VZA	RMSE [0.0~0.5] K	RMSE [0.5~1.0] K	Max *	Min #
0	0	0	10	180	10	195	44	122	0
0	0	0	10	180	20	241	43	49	1
0	0	0	10	180	30	299	40	16	2
0	0	0	10	180	40	316	33	15	0
0	0	0	10	180	50	337	7	1	12
0	0	0	10	180	60	323	4	5	11
0	0	0	20	180	10	243	42	45	1
0	0	0	20	180	20	295	18	11	0
0	0	0	20	180	30	315	34	14	4
0	0	0	20	180	40	337	20	2	3
0	0	0	20	180	50	359	3	1	15
0	0	0	20	180	60	354	7	19	11
0	0	0	30	180	10	299	39	17	2
0	0	0	40	180	10	316	35	16	0
0	0	0	40	180	20	337	20	2	3
0	0	0	40	180	30	361	2	0	6
0	0	0	40	180	40	364	1	0	3
**0**	**0**	**0**	**40**	**180**	**50**	**373**	**0**	**0**	**26**
**0**	**0**	**0**	**40**	**180**	**60**	**373**	**0**	**1**	**47**
0	0	0	50	180	10	336	8	1	14
0	0	0	50	180	20	359	4	1	16
**0**	**0**	**0**	**50**	**180**	**30**	**360**	**0**	**0**	**14**
**0**	**0**	**0**	**50**	**180**	**40**	**373**	**0**	**0**	**26**
0	0	0	50	180	50	353	2	2	14
0	0	0	50	180	60	365	8	5	69
0	0	0	60	180	10	322	5	4	10
0	0	0	60	180	20	354	8	22	12
0	0	0	60	180	30	373	0	1	10
**0**	**0**	**0**	**60**	**180**	**40**	**373**	**0**	**1**	**47**
0	0	0	60	180	50	365	8	3	69
0	0	0	60	180	60	349	0	21	0

* Max stands for the frequency of the maximum temperature difference, while # Min stands for the frequency of the minimum temperature difference.

According to the above four criteria and the results shown in Table 2, we find that large VZAs with respect to the nadir direction can reduce the fitting error, and several angle combinations of VAA and VZA can be considered as candidates for the optimum groups: ① [(0°, 0°), (0°, 30°), (180°, 50°)]; ② [(0°, 0°), (0°, 40°), (180°, 50°)]; and ③ [(0°, 0°), (0°, 40°), (180°, 60°)] (see highlight in Table 2). Although the combination [(0°, 0°), (0°, 50°), (180°, 60°)] produces the minimum error most often, the number of times the maximum error is obtained (five times) and the large viewing zenith angles make this group impractical. 

Among the three candidates, group ③ cannot be recommended because of its large VZA in the direction (180°, 60°), whose pixel sizes are up to four times the pixel sizes at nadir observation if the sensor’s IFOV (instantaneous field of view) remains constant in every direction. Group ② performs slightly better than group ①. However, the final decision cannot be made without a sensitivity analysis of the errors in the observed data. To investigate the sensitivity of the different angle groups to the DBT errors, artificial temperature noises within [−0.5, 0.5] K and [−1.0, 1.0] K with a uniform distribution are added to the simulated DBT of Equation (1). The three coefficients of the TIR-BRDF model are recalculated and subsequently used to obtain the hemispheric DBT. The output of the TIR-BRDF model is constrained whereby the DBT must reach its maximum value in the solar direction because the sunlit components in this direction have the largest percentage in this hotspot direction.

**Figure 3:**
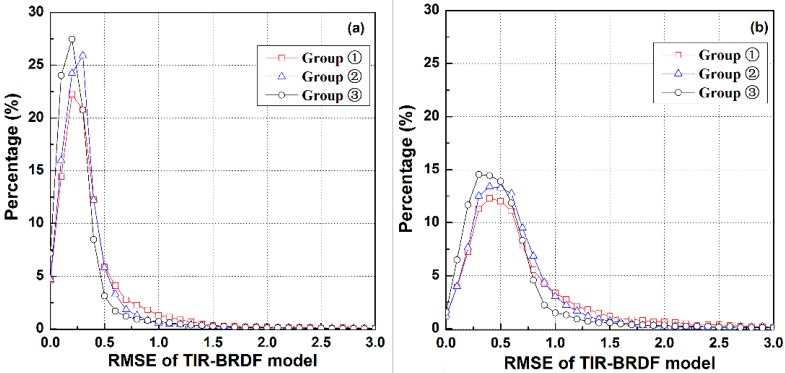
Histograms of the temperature RMSE caused by the three-angle TIR-BRDF model with respect to temperature noise about (**a**) [−0.5, 0.5] K and (**b**) [−1.0, 1.0] K included in the input DBT data, respectively.

Figure 3 shows the histograms of the temperature RMSEs for the three-angle TIR-BRDF model using the angle groups ①–③ with respect to simulated random measurement error in [−0.5, 0.5] K and [−1.0, 1.0] K included in the input DBT data. The figure illustrates that group ③ produces the smallest errors under the two noise conditions, followed by groups ② and ①. The RMSE percentages of groups ①–③ in the range 0.0~1.0 K are approximately 92.4%, 96.6% and 96.3% for the noise level [−0.5, 0.5] K and 80.1%, 88.5% and 91.1% for the noise level [−1.0, 1.0] K. These results indicate that the three groups can produce a temperature error of less than 1.0 K for most cases if the noise involved in the observed DBT data is no more than 1.0 K. However, it is obvious that groups ② and ③ are less sensitive to the noise in the observed data. As stated above, group ③ cannot be used as the optimum viewing angle combination because of the large viewing zenith angle and the occurrence of maximum errors. As a result, group ② should be the optimum group in theory. However, because the pixel size increases with increasing VZA, the ratios of pixel area at VZA = 30°, 40° and 50° to that of nadir are 1.32, 1.70 and 2.40, respectively. It is necessary to use a pixel with a middle area to connect the observations from nadir to large VZAs, especially for heterogeneous surfaces. In this case, the VZAs in group ① produce a more continuous series of pixel sizes (1, 1.3 and 2.4 times the nadir pixel size). In addition, the angle intervals in the slant direction of group ① (*i.e*., 20°) are larger than those of group ② (*i.e*., 10°), which makes group ① less sensitive to the errors in the observation angle in theory. Therefore, according to the single-pattern analysis, we prefer the angle combination of group ① and take this group as the local optimum angle combination for the DBT regression using the three-angle TIR-BRDF model.

### 3.2. Linear-Array Pattern Analysis for Multiple-Viewing Angle Specification

**Figure 4:**
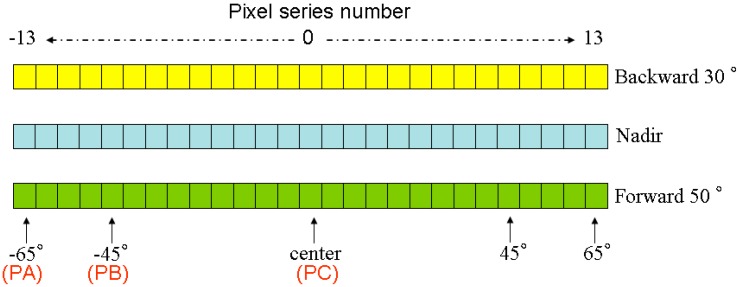
The illustration of three linear-array patterns. The “pixel series number” is the number of each pixel corresponding with Figure 5; the pixels marked with “PA”, “PB” and “PC” will be used for analysis.

Most sensors onboard polar-orbit satellites scan the surface in the crossing-track direction by rotating the mirror toward the target (e.g., MODIS and AVHRR), using the linear-array detectors to monitor the earth at different zenith angles relative to the nadir direction (e.g., Landsat 8 [46]), or taking photos in a central projection manner. In this paper, we use linear-array detectors as an example and suppose that a sensor is equipped with three arrays of detectors to observe the earth at nadir, forward and backward directions and that all detectors have the same spectral and radiative characteristics. Based on the results from the above discussion, we use the angle group ① [(0°, 0°), (0°, 30°), (180°, 50°)] for the array observations and design the detectors (or pixels) for the nadir, forward and backward directions as shown in Figure 4. In this system, each linear array has 27 pixels, and the VZA for the detectors in the nadir array varies in the range of ±65° from left to right with an interval of 5°. If we assume that the sensor travels in the north–south direction and we ignore the influence of the rotation of the earth, the VAA of the left detectors of the nadir array will be 90°, and that of the right detectors will be 270°. The VZA and VAA in the backward and forward arrays can be calculated as

(8) $$\theta_{i,k}=\arctan(\tan\theta_{k}/\tan\theta_{i,0}),\;\phi_{i,k}=\arctan\left(\sqrt{\tan^{2}\theta_{k}+\tan^{2}\theta_{i,0}}\right)$$

where θ*_i,k_* is the VZA of the *i-*th pixel in the backward or forward arrays; θ*_i,0_* is the VZA of the *i-*th pixel in the nadir array; θ*_k_* is the zenith angle of the backward or forward arrays relative to nadir, *i.e.*, θ*_k_* = 30° or 50°; and φ*_i,k_* is the relative angle between the azimuths of the *i-*th pixel in the nadir and off-nadir directions. The absolute azimuth angle will be determined by φ*_i,k_* and the position of the *i-*th pixel in the nadir array.

**Figure 5:**
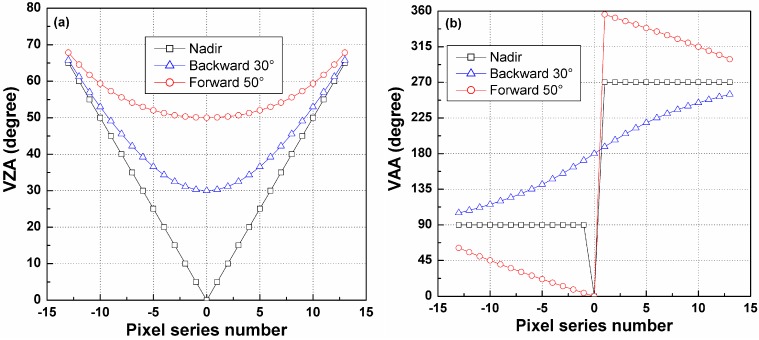
(**a**) Viewing zenith angles and (**b**) viewing azimuth angles of the three linear-array detectors. The pixel series number can be found in Figure 4.

Based on Equation (8), the azimuth and zenith angles for each pixel in the three linear arrays of Figure 4 are calculated and shown in Figure 5. As observed from Figure 5, the differences in the VZA and VAA of the same pixels in the three arrays are large in the middle part of those arrays but decrease as the pixels become closer to the edges of the arrays. Because the angle difference will influence the temperature difference between different viewing directions, even a small difference in viewing angle might cause large errors in the TIR-BRDF model, which will be illustrated in the following discussion.

To verify the performance of the TIR-BRDF model for the array observation pattern and to analyze the influence of the three angles at different locations on the arrays, we first simulated the DBT with varying LAIs and solar positions, subsequently added uniformly distributed noise in [−0.5, 0.5] K to the simulated data, and finally compared the fitted DBT from the TIR-BRDF model with the true DBT from the simulations. The following will discuss the influence of SZA, SAA and LAI on the temperature residual caused by the TIR-BRDF model in the total hemisphere and on the DBT difference in the nadir direction, which is usually considered as the reference direction for the angular normalization of temperature.

#### 3.2.1. Influence of Solar Position

Because the VZA and VAA of each pixel in the arrays are fixed, the solar position impacts the fractions of sunlit soil, shadowed soil and leaves as well as the component temperatures in each pixel. To analyze the sensitivity of the TIR-BRDF model to the solar position, we simulate the DBT by varying the SAA from 0° to 330° with a step of 30°, with the SZA equal to 10°, 30° and 50° for each SAA. A total of 5 LAIs (0.5, 1.0, 2.0, 3.0 and 4.0) are used for each solar position. Artificial noise [−0.5, 0.5] K is added to the simulated DBT. All pixels in the nadir, backward or forward array include the same noise, but different arrays include different noises.

##### A. The Influence of SZA on the TIR-BRDF Model

**Figure 6:**
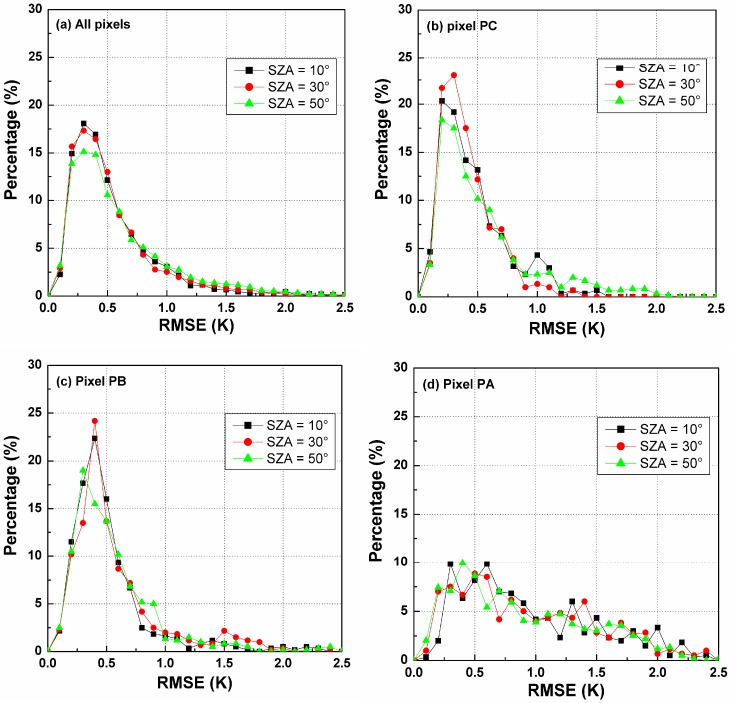
RMSE histograms of DBT difference for different pixels and SZAs. (**a**) is for all pixels in Figure 4; (**b**) is for the pixel PC ; (**c**) is for the pixel PB ;(**d**) is for the pixel PA.

Figure 6 shows the RMSE histograms for the temperature difference produced by the TIR-BRDF model at different SZAs and demonstrates that the percentage of RMSE within [0, 1.0] K for SZA = 10° is larger than for the other two SZAs. A small SZA can lead to a low uncertainty for the estimated DBT and thus less sensitivity to noise. Furthermore, Figure 6b–d are the RMSE histograms of the three pixels denoted by PC, PB and PA in Figure 4, which correspond to VZA = 0°, 45° and 60° in the nadir array, respectively. The case of the pixel PC exhibits the highest robustness to noise (Figure 6b). The pixels (e.g., PA) close to the edge of the array have larger errors in the estimated DBT because, as shown in Figure 5, the differences in their VZAs and VAAs in the three arrays are very small, causing a small variation in the component fractions between the three viewing angles and consequently leading to a small temperature difference in the observed DBT data. Thus, the TIR-BRDF model is shown to be highly sensitive to the noise in the DBT data. Therefore, obtaining a better result from the TIR-BRDF model requires a large difference between the viewing directions.

**Figure 7:**
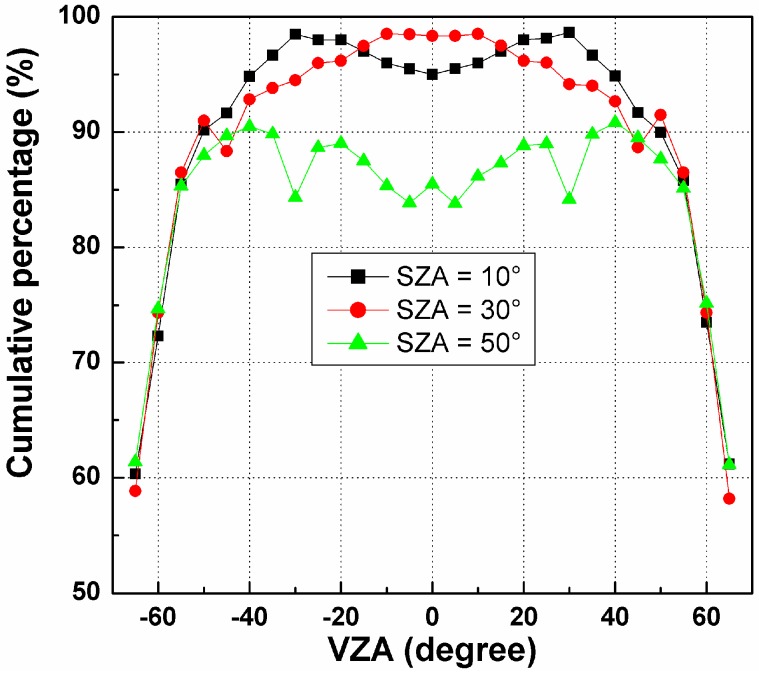
The cumulative percentages of RMSE in [0.0, 1.0] K for different pixels in Nadir array and solar zenith angle (SZA).

Based on the results in Figure 6, the cumulative percentage of RMSE in [0.0, 1.0] K has been calculated for each pixel and SZA, as shown in Figure 7. The VZA of the *x*-axis is the zenith angle of the pixels in the nadir array (see Figure 4). Figure 7 indicates that such cumulative percentages generally decrease with increasing VZA, especially for VZA larger than 45°. For SZA = 10° and 30°, their cumulative percentages are approximately 95% at VZA smaller than 45° and even nearly 100% in the nadir direction. In contrast, this percentage for SZA = 50° is approximately 85%, which is much lower than those of the other two SZAs. Therefore, as observed in Figure 7, an angular observation with a maximum VZA of less than 45° in the nadir array and a relative small SZA is necessary to ensure that the cumulative percentage of the temperature error in the range 0.0~1.0 K caused by the TIR-BRDF model is no less than 85%.

##### B. The Influence of SZA on Temperature Error in the Nadir Direction

**Figure 8:**
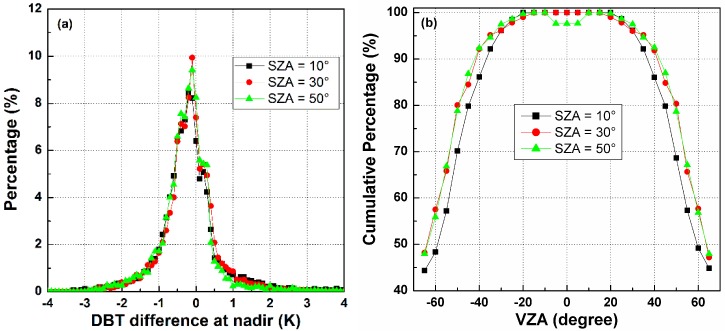
(**a**) The histograms of DBT difference at nadir; and (**b**) the cumulative percentages of DBT difference in [−1.0, 1.0] K for different viewing angles and SZAs.

Once the three kernel coefficients of the TIR-BRDF model are determined, the DBT in any other direction can be estimated in theory. The nadir direction is taken as the reference direction after angular normalization of the temperature, and the DBT is required to be converted from other directions to the nadir observation. Figure 8a shows the histograms of the temperature differences between the normalized DBT at nadir from the TIR-BRDF model and the true nadir value in the simulation. The figure shows that most of the differences are within [−1.0, 1.0] K with a bias of approximately 0.2 K. Similar results are obtained for the three SZAs, and the case of SZA = 30° performs slightly better than that of the other SZAs. Figure 8b shows the cumulative percent DBT difference in [−1.0, 1.0] K for different pixels and SZAs. Similar to Figure 7, the percentages decrease with increasing VZA and are almost distributed symmetrically with respect to the central pixel. For pixels with small VZAs, the TIR-BRDF model performs well at all three SZAs, but its accuracy is reduced for pixels near the edges of linear arrays. These results are similar to those of the previous section and further indicate that the requirement of an angular observation with middle SZA and VZA in the nadir array of less than 45° is necessary for an acceptable result, *i.e.*, more than 85% of cases have a temperature residual error within [−1.0, 1.0] K.

##### C. The Influence of SAA on the TIR-BRDF Model

**Figure 9:**
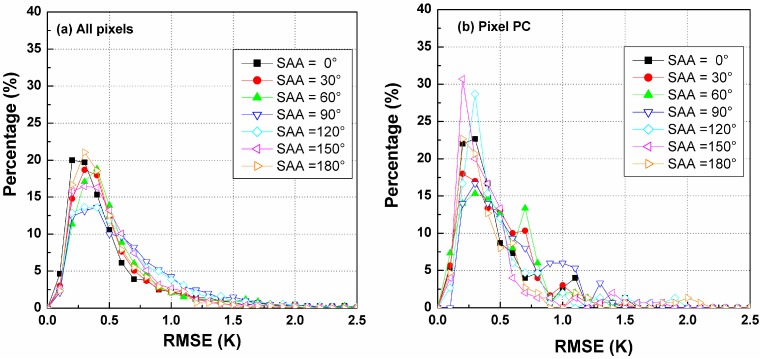

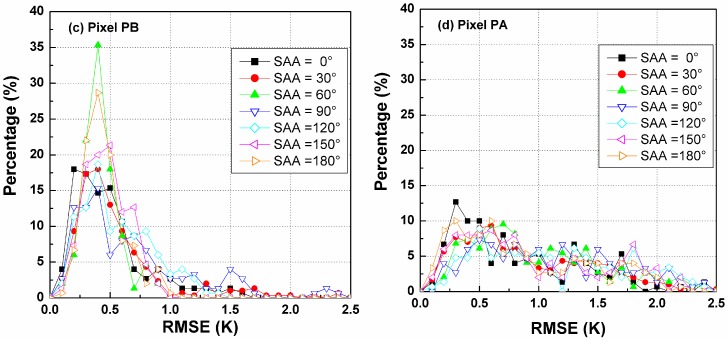
RMSE histograms of DBT difference for different pixels and SAAs.

Similar to Figure 6, Figure 9 shows the histograms for the temperature RMSE produced by the TIR-BRDF model at different SAAs. The values of the pixels (Figure 9d) near the edges of the array exhibit greater uncertainty than the other pixels. It is difficult to decide which SAA is the best for all pixels, but as observed in Figure 9a, the error in the TIR-BRDF model is generally smaller at the solar position that is closer to the VAA (0°~180°). The case of SAA = 90° generally provides the worst results, perhaps because this solar position results in the smallest variation in component fractions and temperatures.

**Figure 10:**
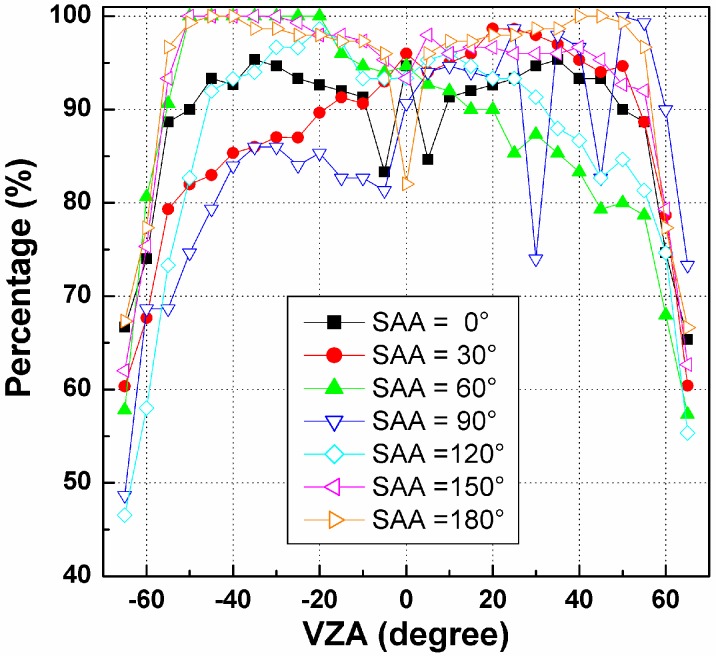
The cumulative percentages of RMSE in [0.0, 1.0] K for different pixels in Nadir array and SAA.

Figure 10 shows the angular variation in the cumulative temperature RMSE percentage in [0.0, 1.0] K. The percentages are not symmetric with respect to the central pixel for SAA = 30°, 60° and 90°, as shown in Figure 7. For example, the percentages on the right-hand side of SAA = 30° are higher than those on the left-hand side, while the percentages on the right-hand side of SAA = 60° are generally smaller than those on the left-hand side. The curve of SAA = 90° varies obviously with the VZA. However, the cases of SAA = 0°, 120°, 150° and 180° are almost symmetric with the central pixel’s location. These different patterns for different SAAs are caused by the difference in the viewing angles in the backward and forward arrays. According to the VAAs of each pixel of the three arrays shown in Figure 5b and the results shown in Figure 9, a cautious conclusion can be drawn that the larger the difference between the solar azimuth and the azimuth angles of array Forward 50° is, the better the result of the TIR-BRDF model will be.

##### D. The Influence of SAA on Temperature Error at Nadir Observation

**Figure 11:**
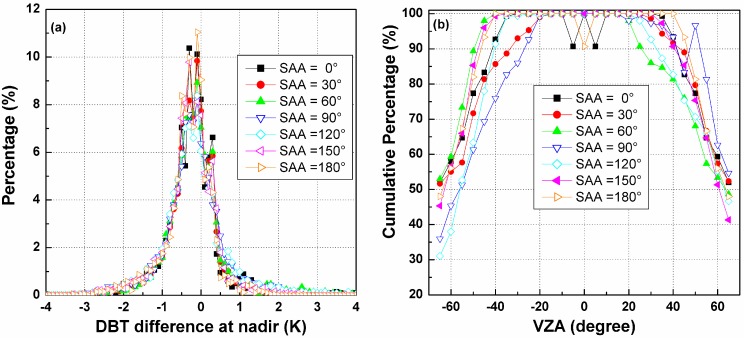
(**a**) The histograms of DBT difference at nadir for different SAAs; and (**b**) the cumulative percentages of nadir DBT difference in [−1.0, 1.0] K for different viewing angles and SAAs.

Figure 11a shows the histograms of the temperature differences between the normalized DBT at nadir from the TIR-BRDF model and the true nadir value in the simulation. The figure illustrates that there is almost no difference among these SAAs. Figure 11b shows the corresponding cumulative percentages with DBT differences in [−1.0, 1.0] K. The results of the SAA = 30°, 60°, 90° and 120° are better on one side compared to the other side, perhaps because of the different viewing zenith angles in the backward and forward arrays. As observed from both Figure 10 and Figure 11, the requirement for an error of no less than 85% of pixels falling in the error range [0.0, 1.0] K requires a maximum VZA in the nadir array that is smaller than 45° for most SAAs but still depends on the SAA.

#### 3.2.2. The Influence of LAI

The LAI affects the gap probability of the canopy and consequently influences the fractions of leaves and soils. Because leaves usually have a lower temperature compared to soil, large LAIs lead to smaller temperatures for the canopy. Using the same methods as above, we have analyzed the influence of the LAI on the result of the TIR-BRDF model with LAI equal to 0.5, 1.0, 2.0, 3.0 and 4.0. The other simulation conditions remain constant with the above discussion.

##### A. The Influence of LAI on the TIR-BRDF Model

**Figure 12:**
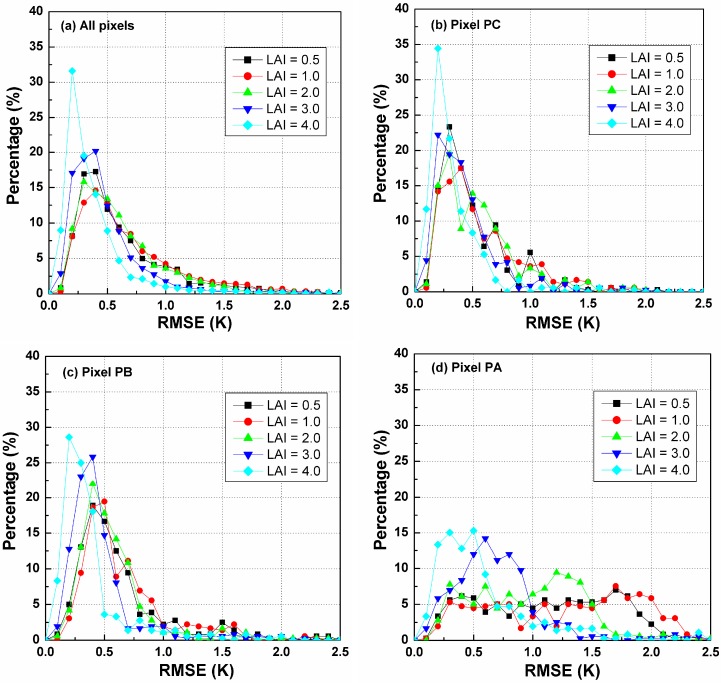
RMSE histograms of DBT difference for different pixels and LAIs. (**a**) is for all pixels in Figure 4; (**b**) is for the pixel PC ; (**c**) is for the pixel PB ;(**d**) is for the pixel PA.

Figure 12 shows the RMSE histograms for different LAIs when there are noises of [−0.5, 0.5] K included in the DBT data, which indicates that the TIR-BRDF model is not very sensitive to the noise for the case of larger LAIs, especially with LAI larger than 2.0. However, the TIR-BRDF model causes a relative larger error for LAI = 1.0 compared to that for LAI = 0.5, possibly because the changes from a soil-dominated canopy (small LAI) to a leaf-dominated canopy (large LAI) degrade the accuracy of the regression process in the TIR-BRDF model.

**Figure 13:**
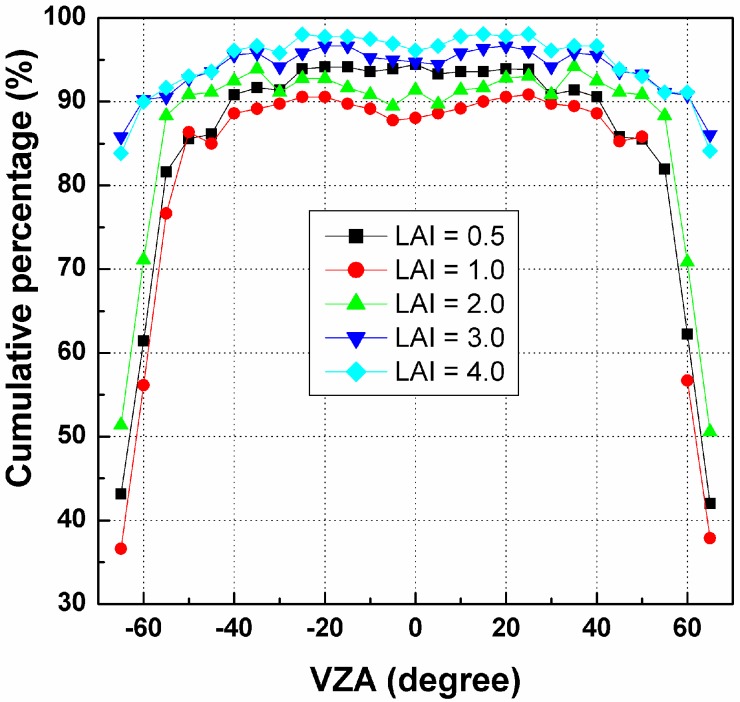
The cumulative percentages of RMSE in [0.0, 1.0] K for different pixels in Nadir array and LAIs.

Figure 13 is the variation in cumulative percentages of the temperature RMSE in [0.0, 1.0] K for the VZAs in the nadir array and for different LAIs. Similar to the influence of the solar position, the pixels closer to the edges of the arrays exhibit smaller cumulative percentages and larger uncertainties, while the pixels in the range [−45°, 45°] produce more reliable results using the TIR-BRDF model. As previously stated, VZAs smaller than 45° are required to obtain reliable fitting of the DBTs using the TIR-BRDF model.

##### B. The Influence of LAI on Temperature Error at Nadir Observation

**Figure 14:**
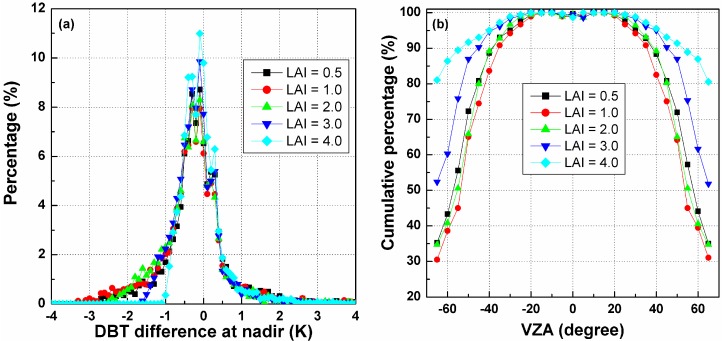
(**a**) The histograms of DBT difference at nadir for different LAIs; and (**b**) the cumulative percentages of DBT difference in [−1.0, 1.0] K for different viewing angles and LAIs.

Figure 14a shows the histograms of the difference between normalized DBTs at nadir from the TIR-BRDF model and the true nadir temperature in the simulation at different SAAs. The figure illustrates, except for the case of LAI = 4, which obviously exhibits the largest percentage in the range of [−1.0, 1.0] K, that no significant difference between the histogram of the other LAIs can be observed. Figure 14b is the variation in cumulative percentages of the nadir DBT difference in [−1.0, 1.0] K with the viewing angles in the nadir array and different LAIs. This figure shows that there are no differences in the cumulative percentage for all LAIs for small VZAs (*i.e*., −20°~20°), but larger LAIs can generally produce higher percentages at large viewing zenith angles. Therefore, the TIR-BRDF model is more reliable for a dense vegetated canopy than for a relatively sparse canopy. The case of LAI = 1.0 produces the smallest percentages perhaps because of the mixed effect described above. Similarly, acceptable cumulative percentages (>85%) for all LAIs require the viewing zenith angle to be less than 45°.

From the above discussion about the three linear-array patterns, we find that the VZA in the nadir array has to be smaller than 45° to ensure that most of the temperature errors from the TIR-BRDF model remain no more than 1.0 K and that the middle SZA and partly and densely vegetated surface can improve the accuracy of the model, and those results from the above single-point pattern and three linear-array patterns have theoretically determined the minimum requirement for the viewing angles and evaluated a local optimum viewing angles for the angular normalization of land surface temperature. These findings can provide some reference for the future development of the multi-angular observation TIR sensors.

## 4. Application of TIR-BRDF Model to WiDAS Data

### 4.1. Airborne WiDAS System

As stated above, to date, no satellite sensors providing multi-angular observation in the TIR domain have been deployed. Therefore, we used an airborne multi-angular image dataset. This dataset was produced using the WiDAS system, which was one of the major airborne sensors used in the WATER synthetic field campaign conducted in the spring to summer of 2008 on the Heihe River watershed in west China [34]. The WiDAS system acquired images using four Charge Coupled Device (CCD) cameras in visible/near-infrared channels (VNIR, 550, 650, 700 and 750 nm) and two broad-band thermal cameras in the mid-infrared (MIR, mainly 3~5 μm, AGEMA 550 from the FLIR company and TIR (mainly 8~12 μm, S60 from the FLIR company) channels. The MIR and TIR cameras were radiometrically calibrated using a man-made blackbody instrument in the temperature range from 273.16 to 358.16 K with an interval of 5 K. The thermal sensitivity of the MIR and TIR cameras were <0.1 K at about 300 K. The spatial resolutions of the MIR/TIR and VNIR images were respectively approximately 7.9 m and 1.25 m at flight height of approximately 1.5 km above the surface.

The cameras in the WiDAS system acquired sequential surface photos at a high frequency during the flight [33], and the overlap between two sequential images was more than 80% in the VNIR channels and more than 85% in the MIR and TIR channels, which meant that the same ground point can almost be simultaneously observed in several sequential images. After performing geometric corrections, a multi-angular dataset was obtained from the collections of the same ground point in the sequential WiDAS images. The WiDAS system was designed to observe the surface in the MIR and TIR channels at a total of seven zenith angles: nadir and backward and forward angles of 10°, 20° and 40°. However, because the time interval between two sequential images was very short (<4 s), the number of observation angles was larger than designed, and the variations in two adjacent VZAs and even VAAs were consequently very small, thereby causing two sequential observations to contain no more angular information about the surface than would only one observation. Moreover, the error in the additional observations might influence the final results of the retrieval, but this dataset provided us an opportunity to analyze the influence of observation angles on the angular normalization of LST using the TIR-BRDF model.

### 4.2. Study Area

**Figure 15:**
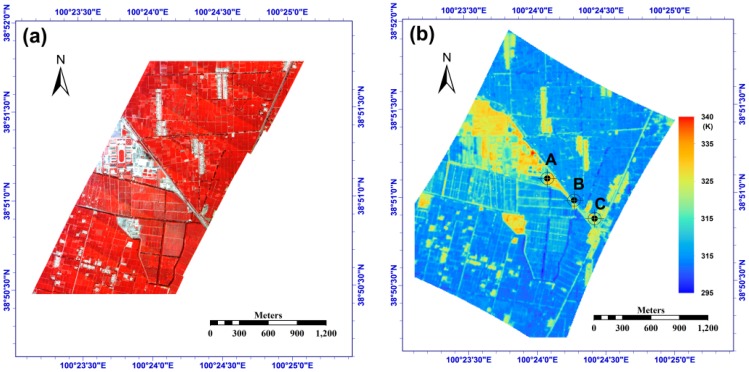

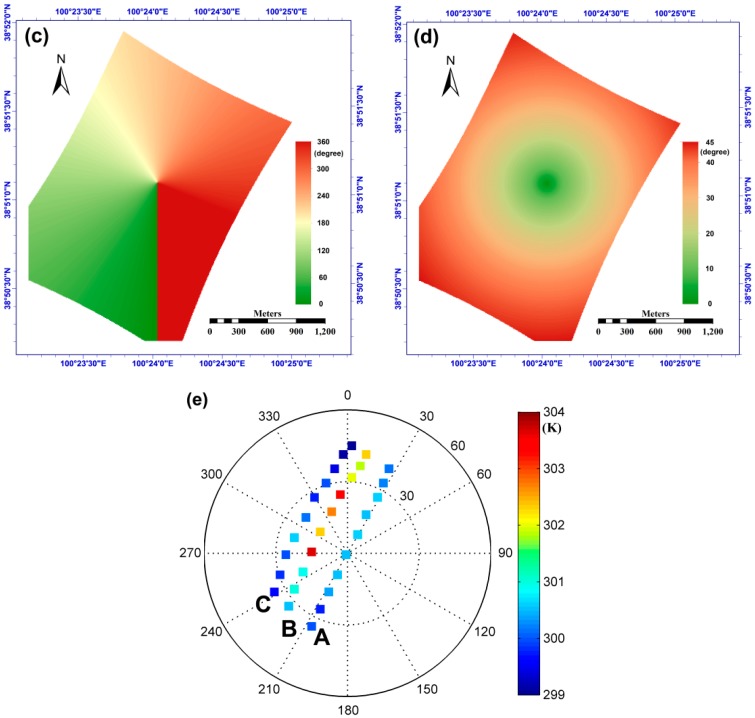
(**a**) Study region (38.84 °N–38.87 °N, 100.39 °E–100.43 °E) VNIR image from the CCD camera; (**b**) The measured brightness temperature of one TIR image collected in the study area; while (**c**) and (**d**) are the viewing azimuth angle and viewing zenith angle, respectively; (**e**) the distribution of viewing direction (VAA: 0°~360° and VZA: 0°~60°) and brightness temperature for the points A, B and C marked in (**b**).

The WiDAS image used in this paper was located in the ZY-YK-HZZ flight zone, which is a typical oasis agricultural area [22,33]. The main land covers included maize, wheat and vegetables. The WiDAS system acquired data over this area on 1 June, 29 June and 7 July 2008. Only the data from 7 July 2008 were used because of its clear-sky conditions and high quality. Figure 15a,b show the VNIR false color image and one TIR image for this area, and these images were acquired at approximately UTC 3:58 (Beijing Time: 11:58) on 7 July 2008. The solar zenith and azimuth angles at that time were approximately 125.7° and 24.4°, respectively. The aircraft flew from north-east to south-west. Because the camera produced a central projection image at every exposure, the VAA and VZA of the image consequently varied alongside the spatial variation in the pixels, as shown in Figure 15c,d. The distribution of the VZA is different sized circles with the same central point. Three points are chosen in the study area, and their locations are marked in Figure 15b: point A is around the flight center line, while points B and C are close to the edge of the image. Figure 15e shows the viewing directions and TIR channel’s brightness temperature of the three points observed in the sequential images of the flight; moreover, the figure indicates that point C has larger VZAs than do points B and C, but the difference in both VZA and VAA between different directions at point A is larger than that of the other two points, which will produce a better result for the angular normalization of LST from the TIR-BRDF model in theory.

### 4.3. Angular Normalization of Temperature

Based on the dataset from the WiDAS system after atmospheric correction using synchronously measured atmospheric profiles (including air temperature, air pressure, and humidity) to drive the MODTRAN code, Ren *et al.* [22] proposed a daytime temperature-independent spectral indices (TISI) method [12] to measure directional emissivities ε(θ*_v_*, φ*_v_*) and directional effective temperatures (*T_e_*(θ*_v_*, θ*_s_*, φ), see Equation (1)) from the observations of both MIR and TIR channels in at least four directions by combining the kernel-driven BRDF model and the TISI method. Their retrieval process generated an effective directional temperature *T_e_* for each direction, whereby the number of directions is equal to the number of observations and is larger than three. Therefore, the three kernel coefficients in the TIR-BRDF model in Equation (4) can be fitted in theory and subsequently used to calculate the temperature at the specified nadir direction. In addition, we also used the temperature RMSE, as shown in Equation (5), to investigate the performance of the TIR-BRDF model.

(9) $$RMSE=\sqrt{\frac{\sum_{k=1}^{N}{\left[T_{e}(k)-T_{e}(k)\prime \right]}^{2}}{N}}$$

where *T_e_*(*k*) is the *k-*th directional effective temperature retrieved from the *k-*th observation, while *T_e_*(*k*)’ is the fitted *k-*th directional effective temperature from the TIR-BRDF model. In addition, to observe the effect of different viewing angle combinations, we used observations from all viewing directions and from two other groups of angular observations with viewing angle differences between two adjacent observations of greater than 8° and 15°. The viewing angle difference ξ*’*, denoted as *delta_angle*, between two directions is calculated as shown in Equation (10), where θ and φ are the VZA and VAA in the *i-*th and *j-*th viewing directions, respectively.

(10) $$\cos\xi \prime =\cos\theta_{i}\cos\theta_{j}+\sin\theta_{i}\sin\theta_{j}\cos(\phi_{i}-\phi_{j})$$

**Figure 16:**
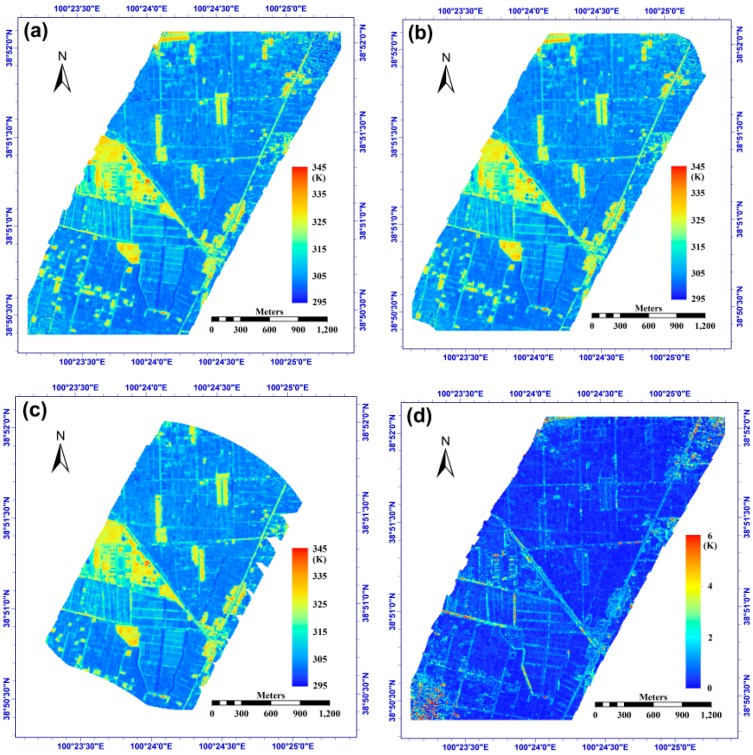

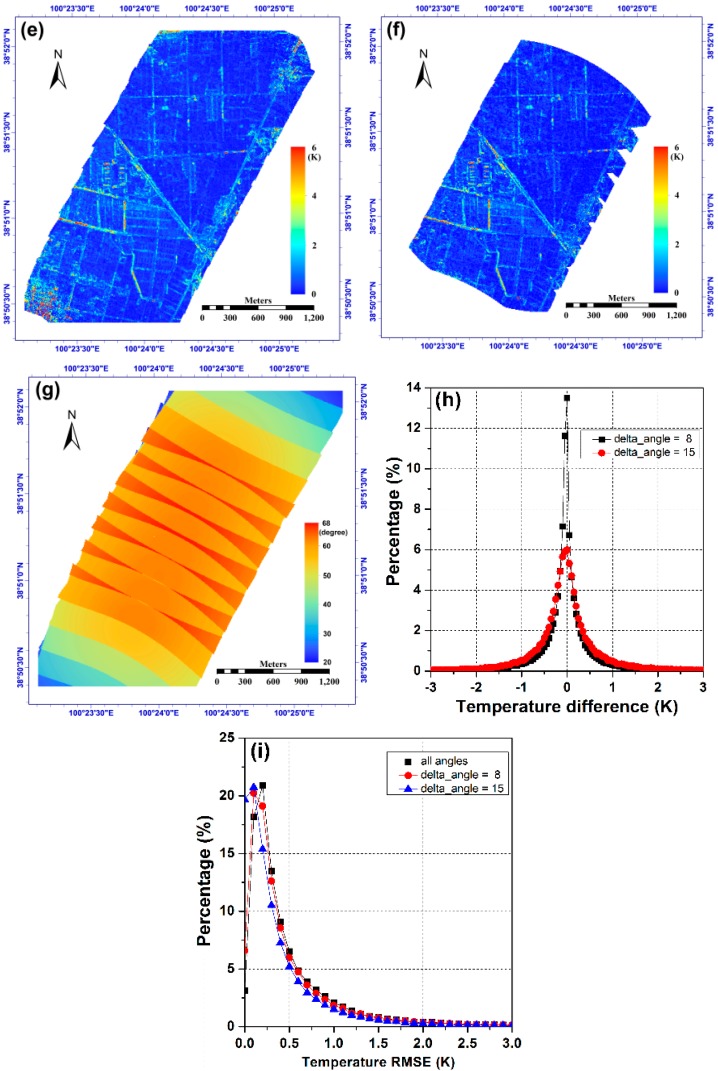
(**a**–**c**) are the effective temperature at nadir (*T_e_-nadir*) calculated from all viewing directions, from the directions with adjacent angle difference larger than 8° (denoted as *delta_angle* = 8°), and from the directions with adjacent angle difference larger than 15° (denoted as *delta_angle* = 15°), respectively; (**d**–**f**) are temperature RMSE in Equation (9) from all directions, *delta_angle* = 8° and 15°, respectively; (**g**) is the maximum viewing angle difference in the study area; (**h**) is the temperature difference between (**a**) and (**b**), and between (**a**) and (**c**); (**i**) is the RMSE distribution of figure (**d**–**f**).

Using the above methods and multi-angular WiDAS data, we performed the angular normalization of temperature from all viewing directions and from the selected directions with *delta_angle* equal to 8° and 15° using Equation (4), and consequently, their directional effective temperatures at nadir (*T_e_*-*nadir*) are shown in Figure 16a–c. From the three figures, we first observe that the temperatures of non-vegetated pixels are generally higher than those of the vegetated pixels and that the results from all observations (see Figure 16a) include the largest number of valid pixels, followed by cases of *delta_angle* = 8° and 15° because the viewing directions in Figure 16a contain those of Figure 16b (*i.e*., *delta_angle* = 8°). Consequently, the viewing directions in Figure 16b contain those of Figure 16c (*i.e*., *delta_angle* = 15°). Although most pixels in the case of *delta_angle* = 15° generally have three or four viewing directions, their angle interval is larger than those of the two other cases. Figure 16g shows the maximum viewing angle differences for all observations from the WiDAS system for each pixel; moreover, the figure shows that the maximum difference can be up to 68° and generally decreases from the center to the edge of the image. The pixels in the top and bottom images have the lowest value (approximately 20°~30°) due to the lack of sufficient sequential images used in this paper. Because of this relatively small viewing angle difference, the top and bottom regions of the study area include no more than three viewing directions as input in Equation (4) after removing viewing directions using the constraint *delta_angle* = 15°.

Furthermore, taking *T_e_*-*nadir*, which is estimated from all viewing directions, as a reference, we calculated the *T_e_*-*nadir* difference between the above three conditions and displayed the temperature difference histograms for *delta_angle* = 8° and *delta_angle* = 15° in Figure 16h, which shows that more than 90% of the temperature differences in the two cases could be found within [−1.0, 1.0] K. In addition, we also found that pixels corresponding to the central portion of the flight exhibit a smaller temperature difference compared to those close to the edge, which is most likely because the viewing angle differences of those central pixels are more significant, as shown in Figure 5 and Figure 15e. In addition, Figure 16d–f are temperature RMSEs (see Equation (9)) calculated from all directions and *delta_angle* = 8° and 15°, respectively; they generally show small differences between each other. In addition, the histograms of their RMSEs, as shown in Figure 16i, indicate that approximately 87%, 88% and 91% of the pixels have a RMSE of less than 1.0 K for the three cases, and the case of *delta_angle* = 15° has a relatively smaller RMSE because of its larger viewing angle interval, which corresponds to larger temperature variations amongst the used viewing directions. From the above discussion, we see that the case of *delta_angle* = 15°, which includes fewer viewing directions, can produce similar or even better results for the angular normalization of temperature using the TIR-BRDF model. Therefore, we can make a cautious conclusion that the TIR-BRDF model (see Equation (4)) can perform well for the angular normalization of LSTs using three or more observations on the same surface as long as the angle difference between the observations is sufficiently large. However, additional viewing directions cannot guarantee a better result because more directions produce more errors in the observed data that will thus reduce the model accuracy in turn.

**Figure 17:**
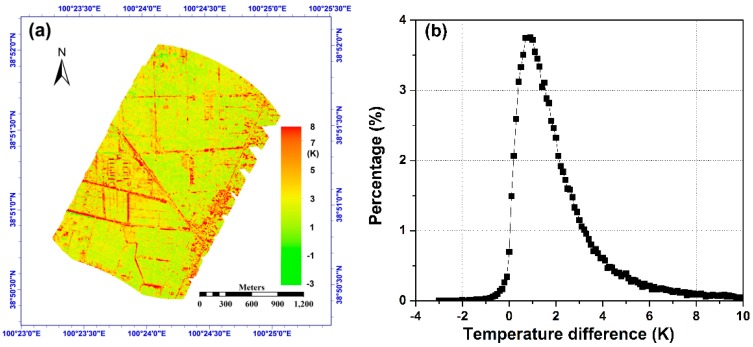
(**a**) The difference of the nadir temperature and the minimum effective temperature of observations; (**b**) is the temperature difference histogram. The case of *delta_angle* = 15° was used for example.

Taking the case of *delta_angle* = 15° for example, Figure 17a shows the temperature difference between the nadir temperature *T_e_-nadir* from the angular normalization and the minimum value of the directional effective temperature *T_e_* under different viewing directions. The color scale of the figure was restricted to 8 K, and pixels with a larger value were set at 8 K for illustration purposes. Meanwhile, Figure 17b shows the temperature difference histogram at a step of 0.2 K. These two figures indicate that *T_e_-nadir* was larger than the minimum temperatures for most pixels, and their differences mainly fell into the range [0.0, 5.0] K. The temperature differences of most vegetated pixels were in the range of [0.5, 2.0] K and those of the non-vegetated pixels were generally larger and even exceeded 8.0 K in certain cases. Such large temperature differences of the non-vegetated pixels may be caused by the angular variation in sunlit and shadow fractions inside those pixels. From this perspective, the angular normalization of temperature is strongly needed for non-vegetated pixels because the angular variation in their temperature can be up to several Kelvins and far from the maximum tolerance of the temperature retrieval accuracy required in practice, *i.e.*, normally 1 K for land surfaces.

**Figure 18:**
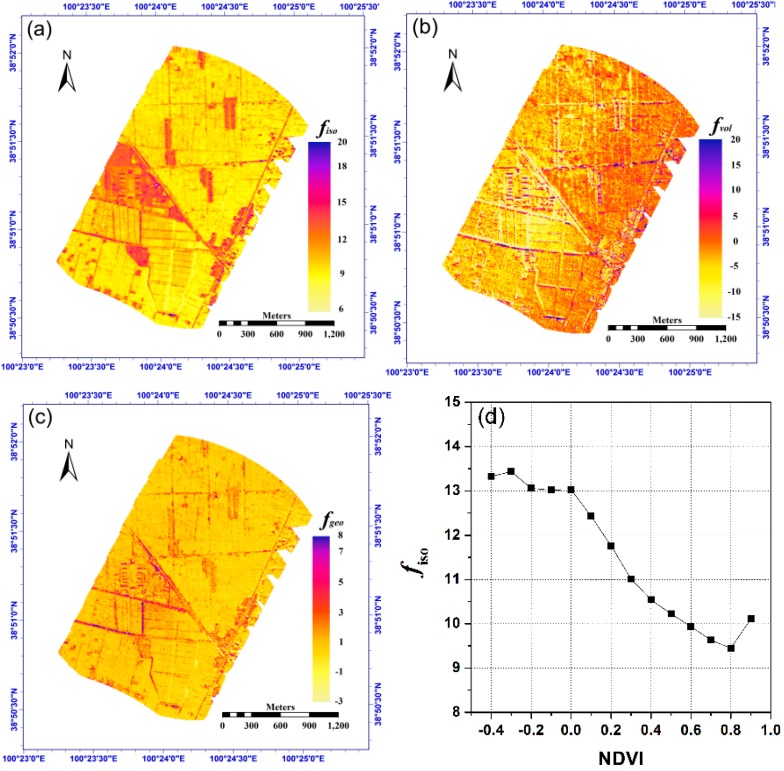

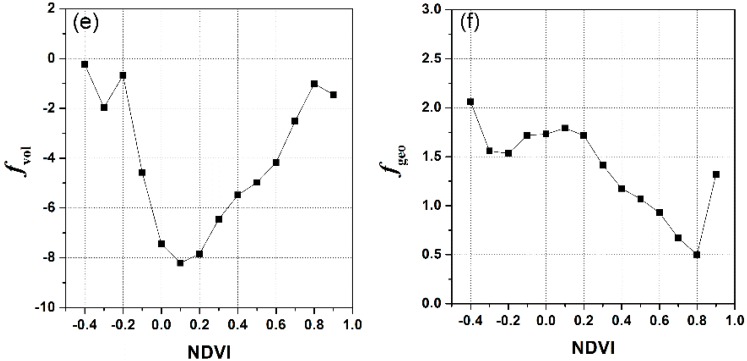
(**a**–**c**) are the kernel coefficients *f_iso_*, *f_vol_* and *f_geo_* in Equation (4), respectively; (**d**–**f**) are the variation of the three kernel coefficients with NDVI in the study area.

Figure 18a–c show the distribution of the three kernel coefficients *f_iso_*, *f_vol_* and *f_geo_* in Equation (4) that were estimated from the case of *delta_angle* = 15°. Compared with the visible image of the study area shown in Figure 15a, we see that the coefficients are highly related to land covers, and the coefficients of vegetated pixels are smaller than those of non-vegetated pixels (e.g., the rural settlement, road and bare surfaces). To investigate this effect, we first used the atmospherically corrected red and near-infrared band reflectance data from the CCD camera to calculate the pixel NDVI and then divided the NDVI into several ranges with a step of 0.1. In each subrange of NDVI, the averages of the corresponding *f_iso_*, *f_vol_* and *f_geo_* are calculated and shown in Figure 18d–f. These figures show that the coefficients *f_iso_* and *f_geo_* generally decreased with increasing NDVI, while *f_vol_* first decreased in the small NDVI range and subsequently increased with increasing NDVI. These results are reasonable because for non-vegetated pixels, taking the rural settlement as an example, their structure makes their angular variation in temperature mainly determined by the fractions of the various components. Therefore, these pixels provided more features in terms of the geometric-optical kernel. In contrast, for vegetated pixels, their internal scattering inside the canopy will contribute more to the thermal radiation observed from different angles and consequently included more features in terms of the volumetric scattering kernel. Because the isotropic coefficient *f_iso_* was related to the temperature (*i.e*., a larger temperature always had a bigger *f_iso_*) and also because vegetated pixels generally corresponding to lower temperatures compared to non-vegetated pixels have large NDVIs, it is reasonable for the coefficient *f_iso_* to decrease with increasing NDVI. Furthermore, we used an NDVI threshold of 0.2 to identify vegetated pixels. A linear relation between NDVI and the three kernel coefficients can be obtained, and this linear relation can be used as a prior knowledge to provide initial guesses for the determination of *f_iso_*, *f_vol_* and *f_geo_* as long as NDVI is known in advance.

## 5. Discussions

Based on the simulated directional thermal radiation from a newly developed model, this paper investigated the minimum requirements for viewing angles for the angular normalization of LST using the TIR-BRDF model and subsequently used the airborne WiDAS multi-angular dataset to validate the TIR-BRDF model and the influence of the number and angle intervals of the viewing directions. Although this paper calculated the component fractions using the parameterization model of the SAILH model always used for homogenous canopy, the new TIR-BRDF model can be also applied to perform angular normalization of LST for heterogeneous surface because the TIR-BRDF is similar to its original kernel-driven BRDF model, and the original model has been successfully used to fit directional reflectance for different land covers to generate surface albedo product (e.g., MODIS global albedo product [47]). However, we noted that the pixel size varies with the viewing angle if the instantaneous field of view (IFOV) remains constant for all viewing directions. As a result, the measurements will consequently include different targets in different viewing directions, especially for heterogeneous and non-isothermal pixels. In addition, the angular variation in temperature is not only caused by changes in the different component fractions but also by the surface variation. However, this effect is inevitable for current satellite sensors that observe the land surface with equal IFOVs, such as the ASTR, whose pixel size at nadir is approximately 1 km but varies to more than 2 km at 55° in the forward direction [48]. This problem, along with the mis-registration between different images, might cause greater uncertainty in the results. To solve this problem, nadir-observed images have to be resampled to the pixel size of the slant observation, or a new multi-angular TIR sensor with variable IFOV should be designed in the future. Furthermore, the lack of more than two directional observations of land surface in the TIR domain by the satellite sensor limits the TIR-BRDF model can only be applied on the airborne images with the current status.

In addition, land surface temperature also exhibits temporal variations, except for angular variation, due to fluctuations in the local meteorological and solar conditions, and this temporal variation is sometimes more significant than the angular variation. However, we ignored this temporal variation and considered the temperature variation to be caused only by changes in viewing angle because, to date, no operational method has been developed to enable the time normalization of temperature from multi-angular observations and also because the time interval between two different observations from the WiDAS system was very short (<4 s). However, the lack of consideration of the temporal variation should be taken into account because it can degrade the accuracy of the TIR-BRDF model.

Besides, because we did not measure ground angular temperature in the study area synchronously with the airborne flight, the validation of the angularly normalized temperature cannot be made to evaluate the performance of the TIR-BRDF model. Therefore, we will pay more attention to this issue in the coming work.

## 6. Conclusions

This paper attempted to evaluate the minimum requirements of viewing angles to enable the angular normalization of land surface temperature by extending the kernel-driven BRDF model to the thermal infrared domain as TIR-BRDF model. First, we developed a new model to simulate the directional thermal radiance (DTR) and directional brightness temperature (DBT) of the canopy, which is assumed to be composed of leaves and sunlit and shadowed soils. The hemispheric DBT simulated from the new method clearly exhibits the hotspot effect. Based on the simulated DBT, the analysis results show that the TIR-BRDF model—which is refined by replacing the bi-directional reflectivity in the primary BRDF model with the directional thermal radiance—performs well in the regression of the hemispheric DBT, thereby leading to an error of less than 0.3 K. If the three kernel coefficients of the TIR-BRDF model are known, the DBT in any other direction can be obtainable in theory. Therefore, the TIR-BRDF model allows us to perform the angular normalization of the DBT. To analyze the performance of this model for angular normalization and to determine the optimum viewing angle combinations, two different patterns were used in the investigation: single-point patterns and linear-array patterns. The single-point pattern indicated the angle combination [(0°, 0°), (0°, 30°), (180°, 50°)] as the local optimum angle combination from numerous three-angle groups because the TIR-BRDF model using the directional thermal radiance under this angle combination provides a robust result and also because the pixel resolution of this angle combination provides a better continuity from fine to coarse scales. Furthermore, we extend this optimum angle combination to three linear-array patterns (nadir, forward 50° and backward 30°), which are assumed to be onboard polar-orbiting satellites and used to observe the earth’s surface at multiple angles as the satellite passes overhead. Investigations of the consistency between the TIR-BRDF model and the solar position and LAIs show that a middle SZA, an SAA along the satellite track direction, and a higher LAI are more appropriate for producing a reliably fitted DBT using the TIR-BRDF model. However, the results depend on the pixel location in the arrays because the closer the pixels are to the edges of the arrays, the worse the results will be. The VZA and VAA differences in pixels in different arrays gradually become smaller as the pixels move from the center to the edge of the arrays. This effect reduces the angular variation in the DBT that is observed by the edge pixels in different arrays and consequently causes the TIR-BRDF model to be more sensitive to the noise in the observed DBT data. Finally, based on the analysis of the temperature RMSE and the temperature difference in the nadir DBT, we determined that the VZA in the nadir array cannot be larger than 45° to ensure temperature RMSEs within [0.0, 1.0] K and the temperature difference in the nadir DBT within [−1.0, 1.0] K for most cases (85%).

Furthermore, we verified the TIR-BRDF model using the multi-angular dataset collected by the airborne WiDAS system and analyzed the influence of the number of viewing directions and angle intervals on the results of the TIR-BRDF model. The results showed that a small number of viewing directions but with large angle intervals can improve the accuracy of the TIR-BRDF model and even produce a better result compared to the use of all viewing angles. This result was most likely due to the large angle interval generally corresponding to large temperature differences between the viewing directions and consequently reducing the residual error in the regression of the TIR-BRDF model. In contrast, additional viewing directions cannot guarantee a better result if their angle intervals are not significant and the errors in the additional data might in turn reduce the accuracy of the final results of the TIR-BRDF model. Based on the angular normalization of the temperature, we found that the nadir temperature was generally larger than that of the slant direction by approximately 0.5~2.0 K for vegetated pixels and up to several K for non-vegetated pixels such as rural settlement. From this viewpoint, it is more important to perform the angular normalization for the non-vegetated surface. In addition, the three kernel coefficients in the TIR-BRDF model were mostly dependent on land covers, and they were linearly related to the pixel NDVI for vegetated pixels. Their linear relation can be used as prior knowledge to provide initial guesses of these three kernel coefficients and can be estimated as long as the NDVI is known.

The findings of this paper may help facilitate the design of future multi-angular observation systems onboard satellites to enable the angular normalization of land surface temperatures and other parameters. However, future work must focus on the pixel size difference caused by the different viewing direction as well as on the temporal variation in temperature, except for the angular variation in this parameter.

## Appendix

**Table A1:** Nomenclatures used in this paper.

Abbreviation	Full Name	Abbreviation	Full Name
ATSR	Advanced Along Track Scanning Radiometer	RAA	Relative Azimuth Angle
AVHRR	Advanced Very High Resolution Radiometer	RMSE	Root-Mean-Square Error
BRDF	Bi-directional Reflectance Distribution Function	SAA	Solar Azimuth Angle
CCD	Charge Coupled Device	SAIL	Scattering by Arbitrarily Inclined Leaves
DBT	Directional Brightness Temperature	SEVIRI	Spinning Enhanced Visible and InfraRed Imager
DTR	Directional Thermal Radiance	SZA	Solar Zenith Angle
FIGOS	FIeld GOniometer System	*Te*-nadir	Nadir Effective Temperature
LAI	Leaf Area Index	TIR	Thermal Infrared
LST	Land Surface Temperature	TIR-BRDF	Thermal Infrared BRDF
MIR	Middle Infrared	TISI	Temperature-Independent Spectral Index
MAOS	portable Multi-Angle Observation System	VAA	Viewing Azimuth Angle
MODIS	Moderate Resolution Imaging Spectroradiometer	VZA	Viewing Zenith Angle
NDVI	Normalized Difference Vegetation Index	WATER	Watershed Allied Telemetry Experimental Research
PARABOLA	Rapid Acquisitions of Bi-directional Observations of Land and Atmosphere	WiDAS	Wide-angle infrared Dual-mode line/area Array Scanner

## References

[1] Hansen J., Ruedy R., Sato M., Lo K. (2010). Global surface temperature change. Rev. Geophys..

[2] Weng Q. (2009). Thermal infrared remote sensing for urban climate and environmental studies: Methods, applications, and trends. ISPRS J. Photogramm..

[3] Li Z.-L., Tang B.-H., Wu H., Ren H., Yan G., Wan Z., Trigo I.F., Sobrino J.A. (2013). Satellite-derived land surface temperature: Current status and perspectives. Remote Sens. Environ..

[4] Li Z.-L., Wu H., Wang N., Qiu S., Sobrino J.A., Wan Z., Tang B.-H., Yan G. (2013). Land surface emissivity retrieval from satellite data. Int. J. Remote Sens..

[5] Sobrino J.A., Li Z.-L., Soria G., Jiménez J.C. (2002). Land surface temperature and emissivity retrieval from remote sensing data. Recent Res. Develop. Geophys..

[6] Qin Z., Karnieli A., Berliner P. (2001). A mono-window algorithm for retrieving land surface temperature from Landsat TM data and its application to the Israel-Egypt border region. Int. J. Remote Sens..

[7] Jiménez-Muńoz J.C., Sobrino J.A. (2003). A generalized single-channel method for retrieving land surface temperature from remote sensing data. J. Geophys. Res..

[8] Wan Z., Dozier J. (1996). A generalized split-window algorithm for retrieving land-surface temperature from space. IEEE Trans. Geosci. Remote Sens..

[9] Du C., Ren H., Qin Q., Meng J., Zhao S. (2015). A practical split-window algorithm for estimating land surface temperature from Landsat 8 data. Remote Sens..

[10] Gillespie A., Rokugawa S., Matsuuaga T., Cothern J.S., Hook S., Kahle A.B. (1998). A temperature and emissivity separation algorithm for Advanced Spaceborne Thermal Emission and Reflection Radiometer (ASTER) images. IEEE Trans. Geosci. Remote Sens..

[11] Hulley G.C., Hook S.J. (2011). Generating consistent land surface temperature and emissivity products between ASTER and MODIS data for earth science research. IEEE Trans. Geosci. Remote Sens..

[12] Becker F., Li Z.-L. (1990). Temperature-independent spectral indices in thermal infrared bands. Remote Sens. Environ..

[13] Jiang G.-M., Li Z.-L., Nerry F. (2006). Land surface emissivity retrieval from combined mid-infrared and thermal infrared data of MSG-SEVIRI. Remote Sens. Environ..

[14] Peres L.F., DaCamara C.C. (2004). Land surface temperature and emissivity estimation based on the two-temperature method: sensitivity analysis using simulated MSG/SEVIRI data. Remote Sens. Environ..

[15] Wan Z., Li Z.-L. (1997). A physics-based algorithm for retrieving land-surface emissivity and temperature from EOS/MODIS data. IEEE Trans. Geosci. Remote Sens..

[16] Chehbouni A., Nouvellon Y., Kerr Y.H., Moran M.S., Watts C., Prevot L., Goodrich D.C., Rambal S. (2001). Directional effect on radiative surface temperature measurements over a semiarid grassland site. Remote Sens. Environ..

[17] Coret L., Briottet X., Kerr Y.H., Chehbouni A. (2004). Simulation study of view angle effects on thermal infrared measurements over heterogeneous surfaces. IEEE Trans. Geosci. Remote Sens..

[18] Lagouarde J.-P., Moreaua P., Irvine M., Bonnefonda J.-M., Coll C. (2004). Airborne experimental measurements of the angular variations in surface temperature over urban areas: Case study of Marseille (France). Remote Sens. Environ..

[19] Minnis P., Khaiyer M.M. (2000). Anisotropy of land surface skin temperature derived from satellite data. J. Appl. Meteorol..

[20] Rasmussen M.O., Göttsche F.-M., Olesen F.-S., Sandholt I. (2011). Directional effects on land surface temperature estimation from Meteosat Second Generation for savanna landscapes. IEEE Trans. Geosci. Remote Sens..

[21] Ren H., Yan G., Chen L., Li Z. (2011). Angular effect of MODIS emissivity products and its application to the split-window algorithm. ISPRS J. Photogramm..

[22] Ren H., Liu R., Yan G., Mu X., Li Z.-L., Nerry F., Liu Q. (2014). Angular normalization of land surface temperature and emissivity using multiangular middle and thermal infrared data. IEEE Trans. Geosci. Remote Sens..

[23] Ren H., Yan G., Liu R., Nerry F., Li Z.-L., Hu R. (2013). Impact of sensor footprint on measurement of directional brightness temperature of row crop canopies. Remote Sens. Environ..

[24] Wan Z., Zhang Y., Zhang Q., Li Z.-L. (2004). Quality assessment and validation of the MODIS global land surface temperature. Int. J. Remote Sens..

[25] Duan S.-B., Li Z.-L., Tang B.-H., Wu H., Tang R. (2014). Generation of a time-consistent land surface temperature product from MODIS data. Remote Sens. Environ..

[26] Deering D.W., Leone P. (1986). A sphere-scanning radiometer for rapid directional measurements of sky and ground radiance. Remote Sens. Environ..

[27] Sandmeier S.R., Itten K.I. (1999). A field goniometer system (FIGOS) for acquisition of hyperspectral BRDF data. IEEE Trans. Geosci. Remote Sens..

[28] Yan G., Ren H., Hu R., Yan K., Zhang W. A portable multi-angle observation system. Proceedings of the IEEE International Geoscience and Remote Sensing Symposium, IGARSS 2012.

[29] García-Santos V., Valor E., Caselles V., Ángeles Burgos M., Coll C. (2012). On the angular variation of thermal infrared emissivity of inorganic soils. J. Geophys. Res. Atmos..

[30] García-Santos V., Valor E., Caselles V., Coll C., Burgos M.Á. (2014). Effect of soil moisture on the angular variation of thermal infrared emissivity of inorganic soils. IEEE Geosci. Remote Sens. Lett..

[31] Sobrino J.A., Soria G., Prata A.J. (2004). Surface Temperature Retrieval from Along Track Scanning Radiometer 2 Data: Algorithms and Validation. J. Geophys. Res..

[32] Noyes E.J., Sòria G., Sobrino J.A., Remedios J.J., Llewellyn-Jones D.T., Corlett G.K. (2007). AATSR land surface temperature product algorithm verification over a WATERMED site. Adv. Space Res..

[33] Liu Q., Yan C., Xiao Q., Yan G., Fang L. (2012). Separating vegetation and soil temperature using airborne multiangular remote sensing image data. Int. J. Appl. Earth Obs. Geoinf..

[34] Li X., Li X.-W., Li Z., Ma M., Wang J., Xiao Q., Liu Q., Che T., Chen E., Yan G. (2009). Watershed Allied Telemetry Experimental Research. J. Geophys. Res..

[35] Li J., Yan G., Mu X. (2010). A parameterized SAILH model for LAI retrieval. J. Remote Sens..

[36] Verhoef W. (1989). Theory of Radiative Transfer Models Applied in Optical Remote Sensing of Vegetation Canopies. Ph.D. Thesis.

[37] Verhoef W. (1984). Light scattering by leaf layers with application to canopy reflectance modeling: The Sail model. Remote Sens. Environ..

[38] Sobrino J.A., Jimenez-Munoz J.C., Verhoef W., Bendikov T.A. (2005). Canopy directional emissivity: Comparison between models. Remote Sens. Environ..

[39] Peng J., Liu Q., Liu Q., Li J., Ma H., Li F. (2011). Kernel-driven model fitting of multi-angle thermal infrared brightness temperature and its applicaion. J. Infrared Millim. Waves.

[40] Roujean J.-L., Leroy M., Deschamps P.-Y. (1992). A bidirectional reflectance model of the Earth’s surface for the correction of remote sensing data. J. Geophys. Res..

[41] Wanner W., Li X., Strahler A.H. (1995). On the derivation of kernels for kernel-driven models of bidirectional reflectance. J. Geophys. Res..

[42] Li X., Strahler A.H. (1992). Geometric-optical bidirectional reflectance modeling of the discrete crown vegetation canopy: Effect of crown shape and mutual shadowing. IEEE Trans. Geosci. Remote Sens..

[43] Jiang G.-M., Li Z.-L. (2008). Intercomparison of two BRDF models in the estimation of the directional emissivity in MIR channel from MSG1-SEVIRI data. Opt. Express.

[44] Tang B.-H., Li Z.-L., Bi Y. (2009). Estimation of land surface directional emissivity in mid-infrared channel around 4.0 um from MODIS data. Opt. Express.

[45] Snyder W.C., Wan Z. (1998). BRDF models to predict spectral reflectance and emissivity in the thermal infrared. IEEE Trans. Geosci. Remote Sens..

[46] Irons J.R., Dwyer J.L., Barsi J.A. (2012). The next Landsat satellite: The Landsat Data Continuity Mission. Remote Sens. Environ..

[47] Schaaf C.B., Gao F., Strahler A.H., Lucht W., Li X., Tsang T., Strugnell N.C., Zhang X., Jin Y., Muller J.-P. (2002). First operational BRDF, albedo and nadir reflectance products from MODIS. Remote Sens. Environ..

[48] Jia L., Li Z.-L., Menenti M., Su Z., Verhoef W., Wan Z. (2003). A practical algorithm to infer soil and foliage component temperatures from bi-angular ATSR-2 data. Int. J. Remote Sens..

